# The contribution of difficulty of an irrelevant task to task conflict

**DOI:** 10.1177/17470218241228709

**Published:** 2024-02-06

**Authors:** Ronen Hershman, Ayelet Sapir, Eldad Keha, Michael Wagner, Elisabeth M Weiss, Avishai Henik

**Affiliations:** 1 Department of Psychology, University of Innsbruck, Innsbruck, Austria; 2 School of Psychology and Sport Science, Bangor University, Bangor, UK; 3 Department of Psychology, The Hebrew University, Jerusalem, Israel; 4 Department of Psychology, Achva Academic College, Beer-Tuvia, Israel; 5 Department of Industrial Engineering & Management, Ariel University, Ariel, Israel; 6 Department of Psychology and The Zelman Center for Brain Science, Ben-Gurion University of the Negev, Beer-Sheva, Israel

**Keywords:** Stroop effect, cognitive control, information conflict, task conflict, Gestalt principles

## Abstract

In the standard colour-word Stroop task, participants are presented with colour words and required to respond to their colour while ignoring their meaning. Two types of conflict might occur in such experiments: information conflict and task conflict. Information conflict reflects the processing of two contradicting pieces of information and is indicated by shorter reaction times (RTs) in congruent than in incongruent trials. Task conflict reflects the additional effort associated with performing two tasks, as opposed to one, and is indicated by shorter RTs in neutral trials than in congruent trials (termed reverse facilitation). While information conflict is commonly seen in Stroop and Stroop-like tasks, task conflict is rarely observed. In the present study, participants were presented with coloured segments that, by applying Gestalt principles, could be perceived as colour words. We found that incongruent trials were slower than congruent trials, suggesting that participants successfully perceived the colour words, which led to involuntary reading. In addition, reversed facilitation was found so that neutral trials (i.e., trials that only consist of one task) were faster than congruent trials (as well as incongruent trials; both consist of two tasks). The presence of both interference from the incongruent trials and reverse facilitation suggests that involuntary reading could also occur in scenarios requiring cognitive effort.

## Introduction

The Stroop task (Stroop, 1935) is one of the most commonly used tasks for examining cognitive control. This task tests the ability to focus on relevant information while ignoring irrelevant information. In the typical Stroop task (MacLeod, 1991), participants are presented with words and instructed to respond to the colours of the ink as quickly and accurately as possible. There are three main conditions in this task: a congruent condition, in which the meaning of the word is the same as the colour of the ink (i.e., the word BLUE presented in blue); an incongruent condition, in which the meaning of the word is different from the presented colour (e.g., the word BLUE presented in green); and a neutral condition, which can be either a non-colour word (e.g., TABLE) or any other stimulus without meaning (e.g., letters string, pseudo-words, symbols, or coloured patches). Typically, incongruent trials are characterised by relatively slow reaction time (RT), congruent trials by relatively fast RT, and neutral trials by RT that often fall somewhere in between. This pattern of RTs results in an interference effect (i.e., slow responses to incongruent trials than to neutral trials) and a small and fragile facilitation effect (Henik et al., 2018; Hershman & Henik, 2019; Kalanthroff & Henik, 2013; Parris et al., 2022).

Recently, there has been a growing interest in different types of conflicts that can be measured in the Stroop task (Littman et al., 2019; Parris et al., 2022), namely *information conflict* and *task conflict*. The information conflict refers to the conflict between two contradicting pieces of information (e.g., the meaning of the stimulus and the colour of the stimulus), and it is commonly examined by the difference between incongruent trials, when the stimulus consists of two contradicting pieces of information, and congruent trials, when the stimulus consists of two matching pieces of information. The task conflict represents a conflict between the relevant and the irrelevant tasks (i.e., responding to the colour of the stimulus and reading the stimulus). Task conflict is often examined by the difference between congruent trials, when the stimulus triggers two possible tasks, and non-word neutral trials, when the stimulus triggers only one possible relevant task. That means that under certain conditions, congruent trials are expected to result in longer RT than non-words neutrals, in an effect that is also known as the reverse facilitation effect (Kalanthroff et al., 2018; Keha & Kalanthroff, 2022).

Studies aiming to investigate the triggering of task conflict have typically examined different types of neutral stimuli (Hershman et al., 2020, 2021; Kinoshita et al., 2018; Levin & Tzelgov, 2016; Monsell et al., 2001; Parris et al., 2022). It has been demonstrated that any stimulus consisting of letters, or something resembling letters, is sufficient to trigger task conflict, including words, pseudo-words, letter strings, and also strings of abstract draws (Hershman et al., 2020, 2021; Monsell et al., 2001). Moreover, it has been suggested that any stimulus that might have morphological/phonological/orthographical meaning might trigger task conflict (Hershman et al., 2021). Consequently, coloured patches were found to result in less task conflict than other types of neutral conditions, such as letter strings and abstract draws, suggesting that task conflict is less likely to occur when using more meaningless neutral stimuli that cannot trigger the process of reading (Hershman et al., 2021).

While information conflict is commonly observed in RT and accuracy, evidence for task conflict is limited to highly sensitive measurements such as brain activation (Aarts et al., 2009; Bench et al., 1993; Carter et al., 1995) or changes in pupil size (Hershman et al., 2020, 2021; Hershman & Henik, 2019, 2020). Interestingly, when task conflict is revealed behaviorally in an RT experiment, it is often under a condition of cognitive-control relaxation (Goldfarb & Henik, 2007; Kalanthroff & Henik, 2013; Kinoshita et al., 2018). Evidence for task conflict, as indicated by reverse facilitation, was found by decreasing the expectation for conflict (Goldfarb & Henik, 2007), deficient control in a stop-signal task (Kalanthroff et al., 2013), or reducing the preparation time (i.e., the cue-target interval) in a task-switching situation (Kalanthroff & Henik, 2014). This suggests that when participants are not prepared for conflict, it is harder for them to avoid performing the irrelevant task, and as a result, responses to conditions that consist of two tasks (both the congruent and incongruent, but not the non-word neutral) are slower, and a reversed facilitation—the behavioural indication of task conflict, is revealed.

In the current study, we aim to test task conflict when the irrelevant task is difficult to perceive. We used congruent, incongruent, and neutral trials, and participants were asked to respond to the colour of the stimuli. In contrast to the standard color-word Stroop studies, our stimuli (see Table 1) did not include explicit words, but rather, the stimuli could be perceived as words by applying the Gestalt principles of closure and figure/ground (Todorovic, 2008). Therefore, we investigated whether the irrelevant task (i.e., the reading task) would still be performed, even when it requires effort.

**Table 1.** Demonstration for the different stimuli (congruent, incongruent, and neutral) conditions in the current experiment.

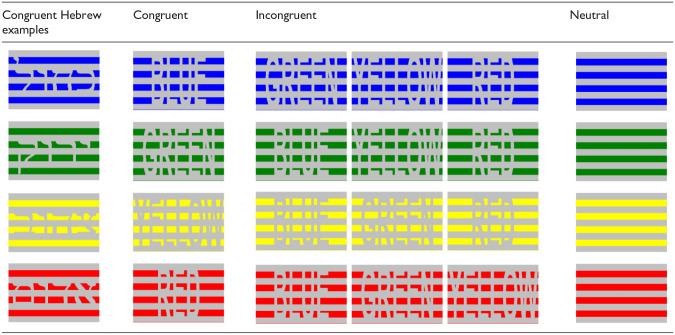

The actual coloured stimuli were in Hebrew (as seen in the Congruent Hebrew examples column). All the presented stimuli in the current study are available here: https://osf.io/sdx2z/?view_only=a5c65347cf8f482497b84b9eaf741948.

Two conflicting predictions can be hypothesised if the stimuli are made hard to perceive as words. Participants may ignore the words and avoid reading them, and thus, no information conflict is expected to be observed; congruent and incongruent trials should be comparable. Indeed, an early study by Gumenik and Glass (1970) found that reducing the readability of the word stimuli by applying a mask reduced the interference effect in a Stroop task. Alternatively, if concealing the words in the stimuli would still trigger reading, a strong information conflict should be seen alongside a task conflict; congruent trials are expected to be faster than incongruent (an indication of information conflict that is caused due to reading of words), but slower than neutral trials (an indication for task conflict).

Why would a stimulus that is hard to process still trigger reading? We would like to offer that any stimulus that resembles words could trigger an involuntary shift of attention towards it (Posner, 1980). If the stimulus is easy to process, such as clearly visible words (MacLeod, 1991), reading will be easy and will not require a lot of resources. In such cases, information conflict will be seen, as in the typical Stroop task, but no task conflict will be expected because the conflict can be easily resolved. However, if the process of reading is triggered with any stimulus that may resemble a word, but the stimulus is hard to process or the word is concealed (as in the current study), reading will occur, which will result in a stronger interference effect. In fact, participants who presumably require increased resources to process the irrelevant task of reading, such as people with dyslexia (Layes et al., 2015) and people responding to stimuli in their second language (Datta et al., 2019; Tzelgov et al., 1992), show greater interference effect in the Stroop task. More importantly, the prediction would be that using stimuli that are hard to be perceived as words will result in a task conflict, as the reading process will require further resources.

The presence of task conflict might suggest that while the activation of the irrelevant task is automatic in terms of being involuntary, the irrelevant task still requires cognitive resources, and therefore, the processing of the irrelevant task can be considered a non-automatic process in terms of cognitive resources (Schneider & Chein, 2003).

## Method

### Participants

Twenty-four students (22 females and 2 males, mean age 23.29 years, *SD* = 1.27) from Ben Gurion University of the Negev participated in the experiment in return for fulfilment of course credit. A power analysis test for the examination of the one-way repeated-measures analysis of variance (ANOVA) with one group and three measurements at a power > 99% with a type 1 error (α = .05) suggested using 22 participants to achieve an effect size of $\eta_{p}^{2}$ = 0.15. Taking into account a possible dropout of participants (due to the chosen platform being an online experiment), the sample size was increased to 24 participants.

The study was approved by the ethics committee of the psychology department. None of the participants reported colour blindness or the presence of a diagnosed neurological and/or psychiatric disorder (including attention disorders or learning disabilities).

### Stimuli

Participants were presented with coloured rectangles, cut into four slices, against a silver (RGB: 192, 192, 192) background. The slices were then cut in a way that by applying the Gestalt principles of closure and figure/ground (Todorovic, 2008), words could be perceived. The colours of the rectangles were blue (RGB = 0, 0, 255), green (RGB = 0, 130, 0), yellow (RGB = 255, 255, 0), or red (RGB = 255, 0, 0), and the colour words were the Hebrew words כחול (kahol—blue), ירוק (yarok—green), צהוב (tzahov—yellow), and אדום (adom—red) in a size of 640 × 740 pixels (see Table 1). In total, three possible congruency conditions were created: congruent trials (i.e., the colour of the stimulus is identical to the meaning of the stimulus), incongruent trials (i.e., the colour of the stimulus is different from the meaning of the stimulus), and the neutral trials (i.e., rectangles without a colour word inside). All the presented stimuli in the current study are available here: https://osf.io/sdx2z/?view_only=a5c65347cf8f482497b84b9eaf741948.

### Procedure

**Figure 1.**
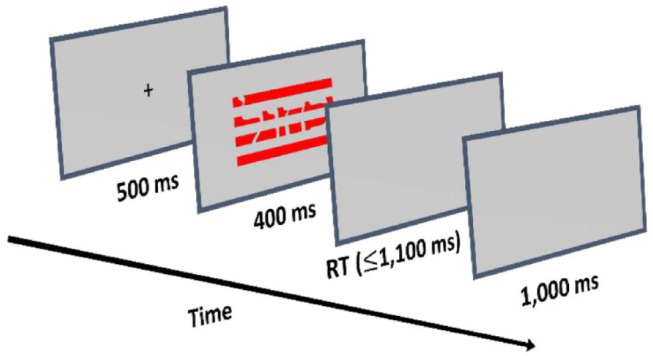
An example of an incongruent trial. Participants had to respond to the ink colour of the stimulus (in this case, red) and ignore the written Hebrew word (in this case, blue).

Participants were tested online by using minnoJS (Zlotnick et al., 2015) on their own devices. The programme required a spacebar response at the beginning of the experiment, ensuring participants used only computers rather than tablets or mobile phones. The experiment included 12 practice trials that were excluded from the analysis. After each practice trial, participants received feedback on their accuracy. Participants were required to achieve at least 85% correct trials in the practice trial to proceed to the experimental part (i.e., at least 10 correct responses). Failure in practice required a repetition of the practice. The experimental part included 432 trials (144 for each congruency condition). At the beginning of each trial (see Figure 1 for a visual demonstration), there was a black fixation cross-presented for 500 ms in the centre of the screen. The fixation was followed by a visual stimulus that appeared on the screen for 400 ms and was followed by a blank screen for a maximum of 1,100 ms or until a keypress. Each trial ended with a 1,000-ms inter-trial interval (ITI) of a blank (silver) screen. The participants were instructed to press as fast as possible the “z” key on the keyboard if the ink colour was blue, the “x” key if the ink colour was green, the “n” key if the ink colour was yellow, and the “m” key if the ink colour was red. RTs were calculated from the appearance of the visual stimulus to the onset of the response.

## Results

One participant was excluded from the analysis because she had less than 70% correct responses in each congruency condition. Analysis of the error rate (*M* = 88.34, *SD* = 6.42) for the other participants (21 females and 2 males, mean age 23.34 years, *SD* = 1.27) suggested that there were no differences between the investigated congruency conditions. $F\left(3,22\right)<1,p=.66,\;\eta_{p}^{2}=0.02,BF_{10}=0.166\equiv$ BF_01_=6.0327.

**Figure 2.**
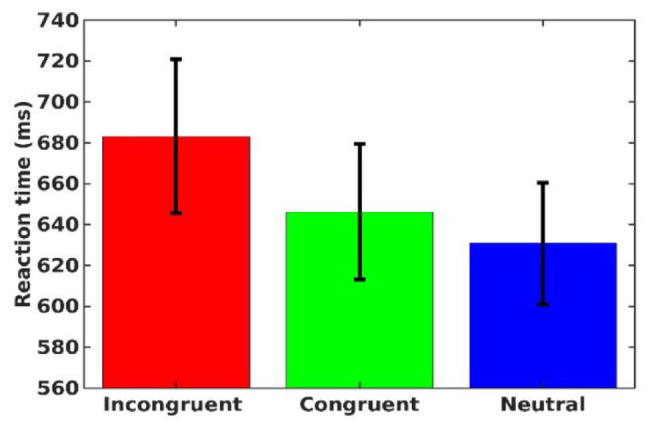
Mean reaction time for each congruency condition. Error bars represent a 95% confidence interval from the mean.

For each participant, mean RTs and standard deviations were calculated separately across all the experimental trials. Then, extremely slow and fast responses were excluded from the analysis (RTs larger or smaller than 2.5 z-scores from the mean of each subject). Mean RTs of correct response trials for each participant in each condition were subjected to a one-way repeated-measures ANOVA with congruency (congruent, incongruent, and neutral) as an independent factor (mean RTs in the various conditions are presented in Figure 2). The analysis produced a meaningful ( $BF_{10}\geq 3$ ) congruency effect $F\left(2,\;44\right)=21.088,\;p<.001,\;\eta_{p}^{2}=0.489,\;BF_{10}\;>10^{4}.$

Post hoc comparisons showed slower responses for incongruent trials than for congruent trials, *F*(1,22) = 20.12, *P*< .001, *BF*_01_ = 157.907, which were slower than neutral trials, $F\left(1,22\right)=9.06,p=.006,\;BF_{10}=7.121$ . Transitively, incongruent trials were slower than neutral trials, $F\left(1,22\right)=24.55,p<.001,BF_{10}=439.094$ . This pattern was robust, as 18 out of the 23 participants showed it.

## Discussion

In the present study, participants were presented with Stroop stimuli and were instructed to respond to their colour as quickly as possible. In contrast to typical Stroop studies (MacLeod, 1991), the stimuli in our study were designed to be difficult to read and, moreover, difficult to identify as actual words. Specifically, the colour words that were used for both congruent and incongruent conditions had the colour of the background and were concealed behind a mask of coloured segments. This coloured mask was used, in fact, as the task-relevant stimulus.

As expected, and in line with most of the previous Stroop studies (MacLeod, 1991), an interference effect was observed with slower responses for incongruent trials than for neutral trials. However, in contrast to the typically observed facilitation effect (i.e., faster responses for congruent trials than for neutral trials), the stimuli used in the present study produced a reverse facilitation effect (i.e., slower responses for congruent trials than for neutral trials). These two effects suggest that our task contains two types of conflict; information conflict and task conflict (Hershman & Henik, 2019; Littman et al., 2019; Parris et al., 2022).

The comparison between incongruent and congruent trials suggests that the observed stimuli caused information conflict (the conflict between the task-relevant information—the colour of the observed stimuli—and the task-irrelevant information—the meaning of the perceived stimuli). This information conflict indicates that the participants perceived, processed, understood, and were influenced by the meaning of the words. In other words, despite the absence of explicitly written words, by using Gestalt principles of closure and figure/ground (Todorovic, 2008), participants were able to read the perceived words. As expected, and in line with other previous Stroop studies (Monsell et al., 2001; Stroop, 1935), a reading of the stimuli caused interference (i.e., slower responses for incongruent trials than for neutral trials) and information conflict (i.e., slower responses for incongruent trials than for congruent trials).

Interestingly, the comparison between congruent trials and neutral trials revealed that there was another conflict—a task conflict—between the relevant task (naming the colour of the stimulus) and the irrelevant task (reading the stimulus). This evidence for task conflict when manual responses were required and with RT as a dependent measure was achieved without decreasing the expectation for conflict (Goldfarb & Henik, 2007), deficient control in a stop-signal task (Kalanthroff et al., 2013), or reducing the preparation time in a task-switching situation (Kalanthroff & Henik, 2014).

The result of this study has implications for the understanding of reading as an automatic process. The presence of information conflict in the current design (i.e., when the irrelevant task is difficult to perceive) suggests that reading is involuntary and cannot be avoided (Monsell et al., 2001; Stroop, 1935). More generally, it suggests that our ability to inhibit the involuntary activation of unwanted processes is limited. In that sense, being an involuntary process, reading can be classified as automatic (Augustinova & Ferrand, 2014; MacLeod, 1991; Stroop, 1935). However, the presence of task conflict also suggests that reading was effortful and required cognitive resources and, therefore, cannot be defined as a fully automatic process in terms of cognitive resources (Schneider & Chein, 2003). Therefore, these findings suggest that reading is an involuntary process that requires cognitive resources. In other words, our results suggest that despite the cognitive resource-intensive nature of the irrelevant task, as evidenced by longer responses to both congruent and incongruent trials than to neutral trials, it still takes place (in our case, the reading occurs).

One can argue that the visual properties of the stimuli (e.g., visual complexity, luminance, number of white pixels, or spatial frequency) might cause the observed pattern. Recently, coloured rectangles, as well as letter strings and abstract draws, were used as neutral stimuli in Stroop and pupillometry studies (Hershman et al., 2021). However, no differences in RT were found among different neutral types. This suggests that the visual properties of the stimulus do not play an important role in the investigation of the Stroop effect, and it is, therefore, unlikely that the visual properties of the neutral stimuli here caused the observed pattern.

Although there was no direct measure of difficulty in our study, the emergence of task conflict, by itself, is an indication that the irrelevant task in our unique stimuli was more challenging than standard words. Furthermore, in a recent study conducted by our group (Hershman, Keha, et al., 2024), we examined the impact of processing difficulty of the irrelevant dimension in the colour-digit Stroop task (Hershman, Beckmann, et al., 2024)^1^ on task conflict. In this study (Hershman, Keha, et al., 2024), participants were presented with numerical stimuli of varying processing difficulty levels, and a gradient of task conflict was observed. That is, as the processing difficulty of the irrelevant dimension increased, there was a corresponding increase in task conflict. This replication, in another Stroop-like task, supports our argument about the impact of the difficulty of the irrelevant task on task conflict.

In addition to the implication this study holds for the reading literature, these results are relevant to a wide range of disorders that are characterised by difficulties in inhibition and cognitive control, such as attention-deficit hyperactivity disorder (Lansbergen et al., 2007), obsessive-compulsive disorder (Hartston & Swerdlow, 1999; Moritz et al., 2008), post-trauma (Aupperle et al., 2012), and addiction (Cox et al., 2006). Our results suggest that reducing the accessibility of an irrelevant task (e.g., a task or an action that should be inhibited) does not improve inhibition abilities and may even increase the demand for cognitive control. Therefore, researchers, as well as therapists who are interested in cognitive control, should take our findings into account.

## Notes

1. In the colour-digit Stroop task (Hershman, Beckmann, et al., 2023), in each trial, participants are presented with a single digit consisting of one to four colours and are required to determine how many colours are presented on the screen while ignoring the numerical value of the digit.
